# Assessing the Influence of Modifiable and Non‐modifiable risk factors on Cognitive and Biomarker Trajectories in Preclinical Alzheimer's Disease: Establishing a Longitudinal Model for Brain Health

**DOI:** 10.1002/alz.084202

**Published:** 2025-01-09

**Authors:** Luciana Maffei

**Affiliations:** ^1^ University of Oxford, Oxford, Oxfordshire UK

## Abstract

**Background:**

The pathogenesis of Alzheimer’s disease (AD) begins 15‐20 years before the onset of clinical symptoms, often referred to as the preclinical stage of AD. It is crucial to understand why some individuals develop AD and although biological, psychological, and social factors have been established, their influence in the very early stages of disease trajectories is somewhat unclear. Longitudinal studies are key to understanding how and when these risk factors determine brain health as well as the risk for developing AD later in life.

**Method:**

This study will extract data from multiple DPUK and non‐DPUK longitudinal datasets containing lifestyle, psychological, environmental, social, and biological data from individuals as young as 18. The study will assess the relationship between a diverse range of biopsychosocial risk factors in the preclinical stage of AD and conduct machine learning statistical analyses to determine which and when these factors interact with biomarker activity contribute to disease risk.

**Results:**

Results are expected to demonstrate a significant interaction between biopsychosocial risk factors and longitudinal biomarker activity in participants <55. Results are also expected to identify the age of biomarker change along with key predictors of initial disease trajectory.

**Conclusion:**

Results from these longitudinal datasets may help with more accurate and earlier prediction tools across various populations and provide rationale to incorporate less understood psychosocial factors into these tools. The analysis will also inform the significance of modifiable factors so individuals can undertake their own preventative methods to reduce AD risk. The study may also have important implications for clinical trial design and promote healthy ageing.

